# Prevalence and Clinical Characteristics Associated with Pulmonary Hypertension in African-Americans

**DOI:** 10.1371/journal.pone.0084264

**Published:** 2013-12-16

**Authors:** Gaurav Choudhary, Matthew Jankowich, Wen-Chih Wu

**Affiliations:** 1 Vascular Research Laboratory, Providence VA Medical Center, Warren Alpert Medical School of Brown University, Providence, Rhode Island, United States of America; 2 Department of Medicine, Warren Alpert Medical School of Brown University, Providence, Rhode Island, United States of America; Indiana University School of Medicine, United States of America

## Abstract

**Background:**

Pulmonary hypertension (PH) is associated with increased mortality and morbidity. It is frequently associated with cardiopulmonary diseases that are prevalent in African Americans (AAs). However, the prevalence or determinants of PH in the AA population is not known.

**Methods:**

We conducted a cross-sectional study to estimate the prevalence of PH (defined as trans-tricuspid gradient ≥ 35 mm Hg) and associated clinical characteristics in AAs using the Jackson Heart Study cohort (n=3,282) who underwent echocardiography and had a measurable trans-tricuspid regurgitant jet. Echocardiography is frequently used for screening for PH despite its limitations in estimating accurate PA systolic pressures. Overall and age-adjusted gender-specific prevalence were estimated and modified Poisson regression was used to identify independent clinical, spirometric, and echocardiographic characteristics associated with PH.

**Results:**

The mean age of the study population was 56.1 ± 12.6 years with 67.5% female. The prevalence of PH was 6.8%, with higher prevalence in female AAs (age-adjusted prevalence: Men 4.9%, 95% CI 3.6-6.2%; Women 7.7%, 95% CI 6.6-8.8%). Pulmonary hypertension prevalence increased with age (Prevalence Ratio: 10.0, 95%CI 4.0-25.1, >65 versus <45 years old), presence of obesity, higher pulse pressure, diabetes, obstructive or restrictive spirometry pattern, and severe left heart valvular disease. Also, PH was significantly associated with left atrial size and left ventricular ejection fraction.

**Conclusions:**

Pulmonary hypertension is prevalent in AAs, more in women than in men. The identified cardiopulmonary risk factors that increase the prevalence of PH may assist in diagnosis and management of these at-risk subjects in the AA population.

## Introduction

Pulmonary hypertension (PH) is associated with increased morbidity and mortality [1,2]. Left heart disease [1,3–5] and chronic pulmonary diseases [6–8] are well-recognized risk factors for PH and are prevalent in the African-American community[9,10]. Data from the National Vital Statistics System suggest a progressive increase in the age-standardized death rates associated with PH as a contributory cause of death, from ~4.5% to 7.3% (from 1980 to 2002) in African Americans (AAs) compared to no substantial change ~5-5.5% in Caucasians [11]. The age-adjusted death rate associated with PH in AAs was the highest reported for any racial group [11]. PH therefore appears to be an important health problem in the AA community. However, neither the prevalence nor the determinants of PH are known in the AA population. 

Pulmonary hypertension is defined as a mean pulmonary arterial (PA) pressure of greater than 25 mm Hg and requires a right heart catheterization for its diagnosis, which is not feasible or practical to perform in population studies. Despite its limitations and inability to accurately assess mean PA pressure at the individual level, Doppler echocardiography is frequently used as a screening tool for PH in populations to estimate the PA systolic pressure [1]. Hence, we used available echocardiography data from a large community-based cohort, the Jackson Heart Study (JHS), to estimate the prevalence of PH (defined as a trans-tricuspid gradient of 35 mm Hg as measured by Doppler echocardiography) and associated demographic and clinical characteristics in AAs. 

## Methods

We conducted a cross-sectional study using the JHS cohort. The conduct of the JHS was approved by the University of Mississippi Medical center Institutional Review Board. The participants gave written informed consent to participate in the research study. The current analysis of the JHS data was approved by the Providence VA Medical Center Institutional Review Board. The Providence VAMC Institutional Review Board waived the requirement for informed consent for this analysis, as the data available to the authors did not contain identifiable information.

### Population

The JHS is a longitudinal population-based cohort study that recruited AA participants residing in Jackson, MS [12]. Participants were enrolled from each of 4 recruitment pools: random, 17%; volunteer, 22%; currently enrolled in the Atherosclerosis Risk in Communities (ARIC) Study, 30%; and secondary family members, 31%. Recruitment was limited to non-institutionalized adult AAs 35-84 years old, except in the family cohort where those 21 to 34 years of age were eligible. The final cohort of 5,301 participants includes 6.59% of all AA Jackson metropolitan statistical area residents aged 35-84[13], who answered predefined questionnaires, and underwent echocardiographic evaluation and spirometry at the time of first exam in 2004. The cohort used for the current study included participants that had echocardiography data available (n= 5,076). Of these subjects, 3,282 (65%) had measurable tricuspid regurgitant (TR) velocity and were used for analysis. Table S1 shows the characteristics of subjects with measurable TR jets compared to the ones without available TR jet. 

#### Outcome

The main outcome is presence of PH, as defined by a trans-tricuspid gradient of ≥ 35 mm Hg, and a pulmonary artery systolic pressure (PASP) of 38-40 mm Hg conservatively assuming a Right Atrial (RA) pressure of 3-5mm Hg [14,15].

#### Exposure & Candidate Determinants

Since there is a paucity of literature on determinants of PH in AAs, we did not have a pre-defined main exposure in the study, but rather investigated a group of demographic, clinical and cardiopulmonary function variables related to the two most common forms of PH (secondary to left heart disease -Group 2 PH; and secondary to lung disease -Group 3 PH) [16] in the general population as potential candidate factors. 

 In order to assess for relationship with Group 2 PH, we chose clinical variables that may be related to increased left atrial pressure [17,18]: coronary heart disease, diabetes, hypertension, a high pulse pressure, body mass index, and severe mitral or aortic valvular disease. The variables used to explore the associations with Group 3 PH included history of chronic lung disease, smoking status, and spirometry measurements [6,17,18]. 

#### Clinical Variables

Coronary heart disease was considered as present when the subject reported a history of myocardial infarction, abnormal stress test, prior coronary artery bypass graft surgery or prior coronary angioplasty. Presence of diabetes was defined as a history of diabetes, use of diabetes medications, HgbA1c ≥ 6.5, or a fasting blood glucose ≥ 126 mg/dL. Presence of systemic hypertension was defined as subject having a systolic BP ≥ 140 mm Hg, or a diastolic BP ≥ 90 mm Hg, or taking anti-hypertensive medications. Pulse pressure was calculated as the difference between systolic and diastolic BP measurement. Body mass index (BMI) was categorized as normal (<25 kg/m^2^), overweight (25-<30 kg/m^2^) and obese (≥30kg/m^2^). Severe mitral or aortic valve disease was considered as present if the qualitative assessment by echocardiography showed presence of severe mitral regurgitation, mitral stenosis, aortic regurgitation, or aortic stenosis. 

Chronic lung disease was considered present if the subjects responded to the question “Has your doctor or health professional ever said you have chronic lung disease, such as bronchitis or emphysema?” in the affirmative. Cigarette smoking status was derived from interview and categorized as never smoker (one who reported having smoked less than 400 cigarettes in one’s life), former smoker (smoked >400 cigarettes but not currently smoking), and current smoker. 

#### Spirometry Measurements

Detailed spirometry procedures for the Jackson Heart Study, including quality control procedures, are available online [19]. Spirometry was performed by certified trained technicians using a dry rolling seal spirometer (Occupational Marketing, Houston TX) connected to a computer. Spirometers were calibrated and leak-tested daily using a 3-liter calibrating syringe. Forced vital capacity maneuvers were performed in the sitting position except in obese (defined for this purpose as BMI>27) subjects; in these subjects, the forced vital capacity maneuver was performed in the standing position. At least three, but no more than five, full forced vital capacity maneuvers were to be obtained on each participant, two of which were to match closely. The "best" maneuver was the one with the highest sum of FVC + FEV1. Technicians received extensive quality control feedback, including immediate real-time computer-based feedback during testing. 

 Predictive equations derived from the NHANES III data for AA men and women were used to calculate percent-predicted FEV1 and FVC [20].. The FEV1 in liters divided by the FVC in liters from the best spirometry maneuver was used to define the FEV1/FVC ratio.

Spirometry measurements used include percent-predicted forced expiratory volume in 1 second (FEV1 % predicted) and forced vital capacity (FVC % predicted). Based on the spirometry measurements, subjects were considered to have an obstructive pattern if the FEV1/FVC ratio was < 0.7, or a restrictive pattern if the FEV1/FVC ratio was ≥ 0.7 and the FVC was <80% predicted. Subjects that had neither an obstructive nor a restrictive pattern were considered as having normal spirometry.

#### Echocardiographic parameters

Detailed echocardiography procedures are available online[21]. Briefly, echocardiograms were recorded by trained sonographers and interpreted by experienced cardiologists in the Echocardiography Reading Center located at the University of Mississippi Medical Center. Standard echocardiographic views were obtained and measurements performed by the interpreting physician who was blinded to the participants’ clinical data [22]. No tissue Doppler measurements were performed. The echocardiography data used for this current study included tricuspid regurgitant (TR) jet peak gradient (trans-tricuspid gradient), semi-quantitative ejection fraction (to nearest 5%), left atrial (LA) diameter (measured on 2D images at end-systole), and pulmonary artery acceleration time (PAT). Left atrial diameter was indexed to height to adjust for body habitus. All measurements used were performed in 2D images. Valvular disease was qualitatively graded.

Given that echocardiographic parameters reflect the underlying pathophysiologic pathway linking left heart disease to PH, these parameters were analyzed separately from the demographic and clinical variables to explore the association of echocardiographic parameters with PH. Left ventricular (LV) systolic function was assessed with ejection fraction (EF) and, in the absence of availability of contemporary Doppler indices to grade diastolic dysfunction [23], we chose left atrial (LA) size as a surrogate for presence of significant diastolic dysfunction. Additional diastolic parameters used included trans-mitral early (E) and late (A) filling velocity that reflect the LA-LV pressure gradients in early and late diastole. 

#### Statistical Analysis

We first plotted the frequency distribution of the trans-tricuspid gradients in the study cohort. Next, we estimated the prevalence of PH and stratified the results based on age groups (<45, 45-54, 55-64 and ≥65) and gender and estimated the age-adjusted prevalence of PH in men and women. We compared the baseline clinical characteristics, pulmonary function and echocardiographic parameters between the PH and non-PH groups using Student’s test and Chi-square analysis, for continuous and categorical variables, respectively.

 We first used a modified Poisson regression model to estimate the prevalence ratios and confidence intervals using robust error variances [24] that included the demographic and clinical variables previously described: age groups, gender, BMI category, pulse pressure, hypertension, diabetes, coronary heart disease, severe mitral or aortic valvular disease, history of chronic lung disease, and the spirometry patterns (obstructive, restrictive or normal). Data was 94% complete for all covariates included in the model. Missing data (6% of the study population) ranged from 0.09% (Body Mass Index) to 4.5% (spirometry data). Missing data were imputed based on 10 sets of simulated values generated from non-missing variables using the multiple imputation method in STATA (StataCorp, College Station, Tex)[25]. Analyses were performed on each of the 10 data sets completed with imputed values, and then combined using Rubin's combination rules to consolidate the individual estimates into a single set of estimates using the MI estimate command in STATA[26]. Next, we performed a sensitivity analysis using the same multivariate model to estimate prevalence ratios using a higher trans-tricuspid gradient to define pulmonary hypertension (trans-tricuspid gradient of 45 mm Hg).

As a separate exploratory analysis on the potential mechanistic associations for Group 2 PH, we used the modified Poisson model to perform age and gender adjusted analyses to assess the relationship of echocardiographic systolic and diastolic parameters with presence of PH. All analysis was performed using STATA/SE version 11.2 software (StataCorp LP, College Station, TX). A 2-sided p- value of < 0.05 was considered significant.

## Results

### Prevalence of Pulmonary Hypertension

**Figure 1:**
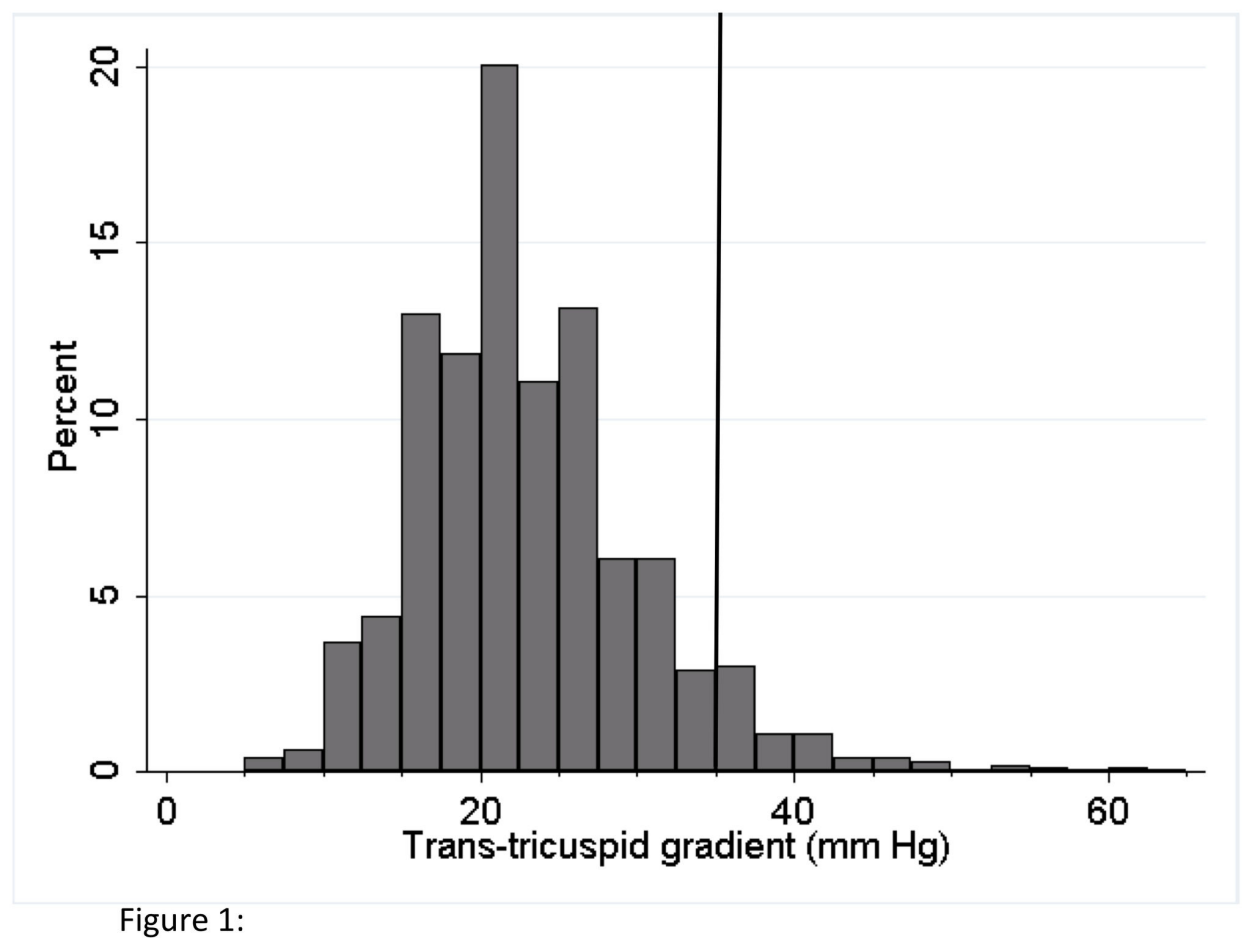
Distribution of trans-tricuspid gradients in study cohort. Vertical line indicates a gradient of 35 mm of Hg that was used as a cut-off for definition of pulmonary hypertension.

**Figure 2:**
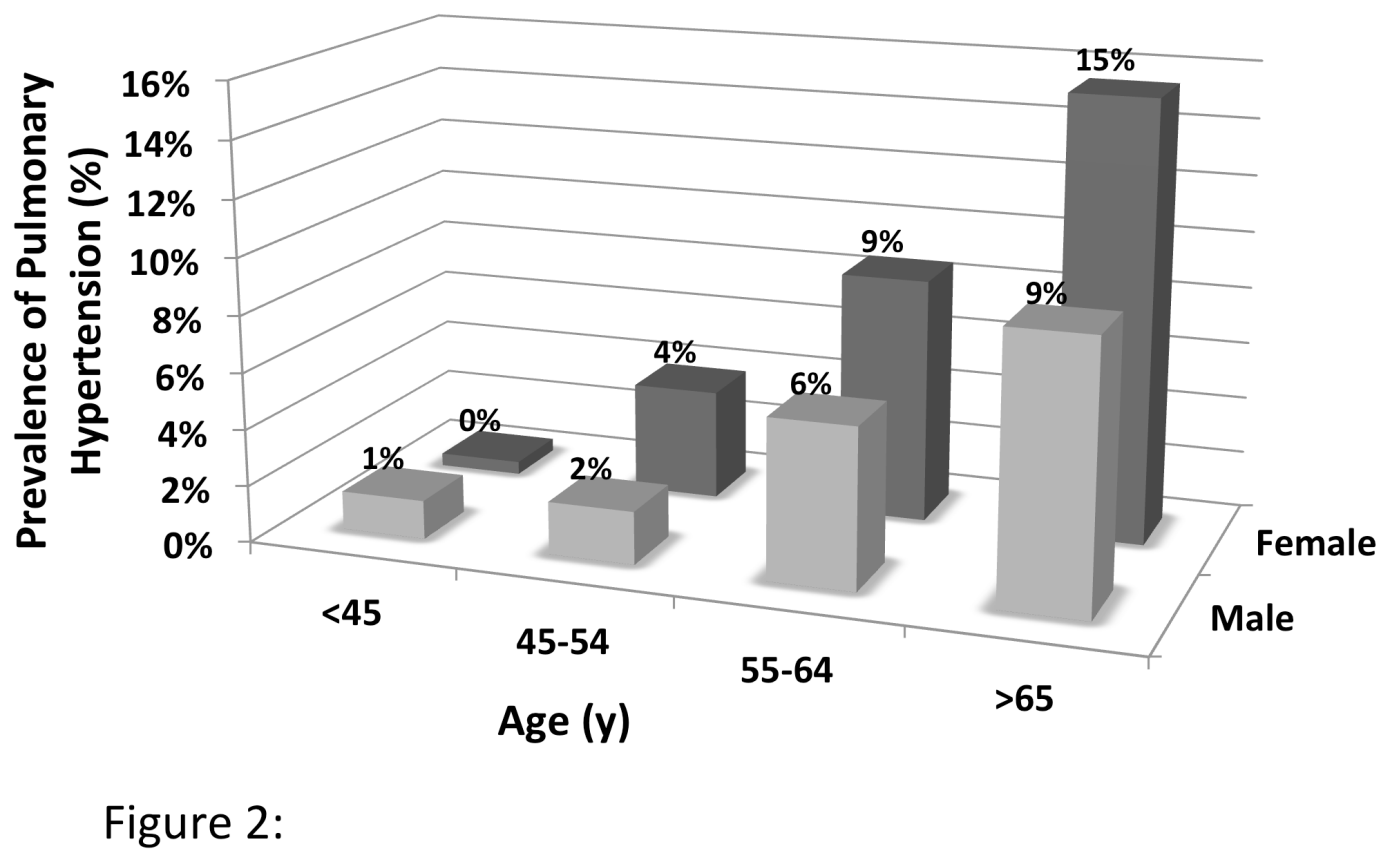
Prevalence of pulmonary hypertension based on age groups and gender.

Figure 1 shows the distribution of trans-tricuspid gradient in our study cohort. The median trans-tricuspid gradient in the cohort was 22 mm Hg (interquartile range: 18 mm Hg). The overall prevalence of PH, defined as a trans-tricuspid gradient ≥35 mm Hg, in the study cohort was 6.8%. The mean trans-tricuspid gradient in the subjects with no PH was 21.6 ± 5.6 mm Hg compared to 40.3 ± 6.0 mm Hg in subjects with PH. The prevalence of PH progressively increased with age (Figure 2) and was higher in female subjects (age-adjusted prevalence: Men 4.9%, 95% CI 3.6-6.2%; Women 7.7%, 95% CI 6.6-8.8%). The highest prevalence of PH was observed in female subjects greater than or equal to 65 y of age at 15.5%, while no subjects less than 35 years of age had PH. 

### Clinical Characteristics of Subjects with Pulmonary Hypertension

Table 1 shows the clinical characteristics of the overall study population and of the groups with and without PH. 

**Table 1:** Baseline Characteristics:.

	**All**	**No PH**	**PH**	
	**(n=3282) Mean ± SD**	**(n=3059) Mean ± SD**	**(n=223) Mean ± SD**	p-value*
**Age**	56.1 ± 12.6	55.4 ± 12.6	65.4 ± 9.4	<0.0001
**Male**	32.5%	33.2%	22.9%	0.002
**BMI Category**				
**Normal**	15.2%	15.6%	10.3%	
**Overweight**	33.7%	34.1%	28.2%	
**Obese**	51.0%	50.3%	61.4%	0.004
**Systolic BP (mm Hg)**	126.5 ± 18.2	125.9 ± 17.9	133.9 ± 21.4	<0.0001
**Diastolic BP (mm Hg)**	78.2 ± 10.4	78.4 ± 10.4	75.6 ± 10.7	0.0001
**Pulse Pressure (mm Hg)**	48.3 ± 15.8	47.5 ± 15.3	58.3 ± 18.9	<0.0001
**Hypertension**	60.9%	59.4%	82.1%	<0.0001
**Diabetes**	24.6%	23.2%	42.6%	<0.0001
**Coronary Heart Disease**	8.3%	7.9%	13.9%	0.002
**Severe Mitral/ Aortic Valvular disease**	0.2%	0.1%	1.8%	<0.0001
**Chronic Lung Disease**	7.0%	6.5%	13.9%	<0.0001
**Smoking History**				
**Never Smoked**	68.1%	68.3%	64.9%	
**Former Smoker**	19.8%	19.6%	23.4%	
**Current Smoker**	12.1%	12.1%	11.7%	0.376
**FEV1 % predicted**	91.7 ± 18.2	92.4 ± 17.7	82.2 ± 21.8	<0.0001
**FVC % predicted**	91.0 ± 18.6	91.5 ± 18.1	84.7 ± 23.8	<0.0001
**FEV1/FVC**	80.2 ± 9.3	80.5 ± 9.1	76.4 ± 10.9	<0.0001
**Spirometry Profile:**				
**Normal**	69.6%	70.9%	50.2%	
**Obstruction**	9.5%	8.9%	18.2%	
**Restriction**	21.0%	20.2%	31.5%	<0.001

^*^ Comparison between PH and no PH groups.

The average age of the study cohort was 56.1 ± 12.6 years and consisted of 67.5% females. Over half of the study population was obese (51%) with a high prevalence of hypertension (61%) and diabetes (24.6%). The majority of the study participants never smoked (68%) and had a normal spirometry profile.

Subjects with PH were older and more likely to be female and to have a higher BMI and pulse pressure than subjects without PH. Cardiovascular comorbidities such as hypertension, diabetes, coronary heart disease, and severe left-sided valvular disease were more prevalent in the PH group compared to subjects without PH. In addition, the subjects with PH had a higher prevalence of reported chronic lung disease, and lower percent-predicted FEV1 and FVC and lower FEV1/FVC ratio than subjects without PH. Consequently, both obstructive and restrictive spirometry patterns were significantly more common in the PH group. A restrictive spirometry pattern was seen in 31.5% of subjects with PH and 20.2% of subjects without PH, and obstruction was seen in 18.2% and 8.9% of PH and non-PH subjects, respectively. On the other hand, the distribution of current and former smokers did not significantly differ between the PH and non-PH groups.

### Echocardiographic Parameters in Subjects with Pulmonary Hypertension

Table 2 shows the echocardiographic parameters of the study cohort. The mean EF of the study cohort was 62.3 ± 7.6%, with a relatively low overall prevalence of reduced EF (2.8% with EF < 50%). Twelve percent of the cohort had a dilated left atrium (LA diameter≥ 4cm). Compared to the subjects without PH, subjects with PH were more likely to have a reduced LVEF; larger LA dimensions; and significantly higher trans-mitral early and late flow velocities, which could represent higher LA pressures. 

**Table 2:** Echocardiographic Parameters:.

	**All**	**No PH**	**PH**	
	**(n=3282) Mean ± SD**	**(n=3059) Mean ± SD**	**(n=223) Mean ± SD**	p-value*
**Echocardiographic Characteristics:**				
**Trans-tricuspid Gradient (mm Hg)**	22.9 ± 7.3	21.6 ± 5.6	40.3 ± 6.0	
**Pulmonary Acceleration Time (msec)**	126.7 ± 32.8	127.4 ± 32.8	116.8 ± 32.4	<0.0001
**Heart Rate**	64.6 ± 11.1	64.5 ± 11.0	66.2 ± 12.6	0.037
**LA diameter index (mm/m^2^)**	18.0 ± 2.5	17.9 ± 2.4	19.3 ± 3.0	<0.0001
**LA dilation (≥4cm)**	12.6%	11.1%	34.2%	<0.001
**Ejection Fraction (%)**	62.3 ± 7.6	62.2 ± 7.3	62.5 ± 11.3	0.65
**LV Ejection Fraction**				
**Normal**	97.2%	97.6%	91.4%	
**Reduced**	2.8%	2.4%	8.6%	<0.001
**E velocity (m/s)**	0.8 ± 0.2	0.8 ± 0.2	0.9 ± 0.3	<0.0001
**A velocity (m/s)**	0.8 ± 0.2	0.8 ± 0.2	0.9 ± 0.3	<0.0001

LA: Left Atrium, LV: Left Ventricle, Normal LV Ejection fraction defined as ≥50%, E: Early Diastolic Transmitral Flow, A: Late Diastolic Transmitral Flow . * Comparison between PH and no PH groups.

### Demographic & Clinical Characteristics Independently Associated With Pulmonary Hypertension

In multivariate regression analysis incorporating age, gender, and clinical cardiopulmonary characteristics, the prevalence ratio of having PH independently increased with age, being female, being obese, or having diabetes, higher pulse pressure, severe aortic or mitral valve disease, chronic lung disease, or an obstructive or restrictive pattern on spirometry. The prevalence ratio for PH associated with restriction (1.76) was similar to that for airway obstruction (2.09), and this association was significant despite the adjustment for body mass index, suggesting the association of PH and spirometric restriction is independent of obesity (Table 3). 

**Table 3:** Association of Demographic and Clinical Characteristics with Presence of Pulmonary Hypertension in Multivariate Analysis:.

	**Prevalence Ratio**	**95% Confidence Interval**
**Age Group (y)**		
**<45**	Referent	
**45-54**	3.47	1.34-8.97
**55-64**	6.67	2.71-16.42
**≥65**	10.02	4.00-25.12
**Male**	0.72	0.53-0.97
**BMI Category**		
**Normal**	Referent	
**Overweight**	1.24	0.79-1.96
**Obese**	1.66	1.08-2.55
**Pulse Pressure (per mm Hg)**	1.01	1.01-1.02
**Hypertension**	1.20	0.85-1.70
**Diabetes**	1.43	1.11-1.85
**Coronary Heart Disease**	1.02	0.71-1.46
**Severe Mitral/ Aortic Valvular Disease**	5.51	2.53-11.99
**Chronic Lung Disease**	1.83	1.26-2.66
**Spirometry Profile**		
**Normal**	Referent	
**Obstruction**	2.09	1.49-2.94
**Restriction**	1.76	1.31-2.35

BMI: Body Mass Index.

Sensitivity analysis using a cutoff of 45 mm Hg for pulmonary hypertension did not change the trend of the point estimates of the prevalence ratios associated with age, being female, being obese, or having diabetes, higher pulse pressure, severe aortic or mitral valve disease, chronic lung disease, or an obstructive or restrictive pattern on spirometry (Data not shown).

### Echocardiographic Parameters Independently Associated With Pulmonary Hypertension

 Age- and gender-adjusted multivariate analysis of available echocardiographic parameters demonstrated that presence of parameters that suggest diastolic dysfunction, such as a higher LA size or higher trans-mitral E velocities, or systolic dysfunction (LVEF<50%) were independently associated with the presence of PH (Table 4). 

**Table 4:** Age- and Gender-Adjusted Association of Echocardiographic Characteristics with Presence of Pulmonary Hypertension in Multivariate Analysis:.

	**Prevalence Ratio**	**95% CI**
**Echocardiographic Characteristics:**		
**Heart Rate (beats/ min)**	1.01	0.99-1.02
**LA Diameter Index (mm/m^2^)**	1.12	1.06-1.19
**LV Ejection Fraction <50%**	2.27	1.40-3.68
**E Velocity (cm/s)**	5.85	2.99-11.47
**A Velocity (cm/s)**	0.58	0.27-1.24

LA: Left Atrium, LV: Left Ventricle, E: Early Diastolic Transmitral Flow, A: Late Diastolic Transmitral Flow.

## Discussion

There are little data regarding PH prevalence in the general population or in AAs. The prevalence of PH of 6.8% in our community-based cohort is similar to the prevalence reported in large populations of mostly Caucasians evaluated by echocardiography for clinical reasons in the INCIPIT study in Italy (TR jet velocity 3m/s: prevalence of 6.6%) [27]. While one study had found AAs to have higher mean PA pressures independent of age, gender, BMI, smoking status, and presence of diastolic dysfunction [28], our findings do not support a higher prevalence of PH prevalence in AAs compared to the previous report based on the Italian cohort.

We report that prevalence of PH increased with increasing age independent of other comorbidities and cardiopulmonary function. Notably, the prevalence ratio of having PH in subjects >65 y was 10.0 times that of those younger than 45y of age. These data are in agreement with the observation in the Olmstead County cohort [1] that demonstrated increasing PA pressures with increasing age and higher age-specific PH death rates in older age groups (11). The higher prevalence of PH in older people may be related to decreasing compliance of the pulmonary arteries with age, as also occurs in the systemic arteries [1]. Our observation of an increasing prevalence ratio of PH with increasing systemic pulse pressure, a marker of systemic arterial compliance, is consistent with this pathophysiological link.

We also show that women have an increased age-adjusted prevalence of PH compared to men. Although idiopathic pulmonary arterial hypertension is more prevalent in women, to the best of our knowledge this is the first study demonstrating the increased prevalence of PH overall in women in a population-based cohort. In contrast, a relationship between gender and PA pressures was not observed in a general population of young adults [28], in normal echocardiograms performed over a decade at Massachusetts General Hospital [29], or in the Olmstead County population cohort [1]. The gender influence on PH in our cohort was independent of age, BMI, and cardiovascular and pulmonary disease, which may suggest a role of race- and gender-specific biologic factors such as estrogen and estrogen metabolites [30–33] and needs further investigation. 

Pulmonary hypertension due to left heart disease (Group II) and associated with chronic lung disease (Group III) are the most commonly observed PH groups in the population [16]. The pathophysiology underlying group II PH is elevated pulmonary capillary wedge pressure as a result of LV diastolic or systolic dysfunction and/or significant left-sided valvular disease. In fact, we showed that known risk factors for increased left atrial pressure such as diabetes[34], obesity[35], and severe left-sided valvular disease were independently associated with PH in this cohort of AAs. Over half of the study population in our cohort had hypertension and about a quarter of them had diabetes. The high prevalence of these comorbidities in AAs highlights the importance of these risk factors as potential targets for the prevention and therapy of PH in the AA population. The independent association of PH with echocardiographic parameters such as left atrial size, a surrogate for presence of high LA pressure in diastolic dysfunction [23], and low EF support the assumption that Group II PH may be prevalent in our study cohort. 

We found that obesity was independently related to PH, an association that has been reported previously for Group 1 PH[31,36] and group 2 PH [37] in other cohorts. The high prevalence of obesity in AAs in our study, at 51%, and in the US overall, at 49.6% (95% CI: 44.5%-54.8%)[38], highlights the public health importance of this relationship. The pathophysiological mechanisms that are commonly attributed to PH in obese individuals are: diastolic dysfunction, obesity-hypoventilation syndrome, and sleep apnea. Since we did not have blood gas analysis, polysomnography, or nocturnal pulse oximetry data in our cohort, we cannot estimate the contribution of sleep apnea or obesity-hypoventilation syndrome to PH in our cohort. Others have reported a high burden of sleep symptoms in the JHS cohort [39], a finding that may reflect the presence of underlying sleep-disordered breathing conditions responsible in part for the observed association. In addition, alternative mechanisms that may mediate the association between PH and obesity include insulin resistance and the pro-inflammatory state that has been associated with obesity [33]. 

Chronic obstructive pulmonary disease is associated with PH (Group 3 PH) [40]. We observed an increased prevalence of chronic lung disease in subjects with PH and that the presence of airway obstruction by spirometry was associated with an increased risk for PH. Presence of PH in COPD portends poor prognosis and may be related to increased exacerbations of this disease[6–8]. This may be of particular importance in our cohort since AAs have a two-fold increased rate of emergency room visits for chronic lung disease and are more likely to be hospitalized for COPD than their Caucasian counterparts[9]. In addition, we report a significant association, of similar magnitude, between a restrictive spirometry pattern[41–45] and the presence of PH. To our knowledge, this is the first study that has associated restrictive spirometry with PH in a large population-based sample. While restriction can result from fibrotic lung disease, a rare disorder associated with PH, the high prevalence of restriction in our sample is unlikely to be explained by pulmonary fibrosis alone. Although we speculate that the restrictive spirometry pattern in our cohort may be related to the highly prevalent obesity [44], we cannot explain the persistent significant association of restriction with PH despite adjustment for known confounding conditions such as obesity, diabetes [41], heart disease [41], and hypertension [41], and this suggests other yet unrecognized mechanisms which merit further investigation. 

Our study has limitations. 1) The main limitation is the cross-sectional nature of the design that limits the potential to establish a causal link between etiologic factors and PH. 2) There was a lack of TR jet on all subjects, similar to what has been observed in other population-based studies[1], and subjects without a TR jet may have been less likely to have PH. Additionally, presence of obesity can make echocardiography technically difficult and this may be one of the factors explaining the higher prevalence of obesity in the cohort without any detectable TR jet. 3) We are unable to validate the presence of PH, as defined by mean PA pressures, in our cohort due to lack of invasive hemodynamics. Although right heart catheterization is necessary for diagnosis and management of PH at an individual patient level, echocardiography remains a useful tool for screening for PH in the population. 4) We did not have echocardiographic parameters that could help us calculate pulmonary vascular resistance or contemporary indices of RV function such as tricuspid annular plane systolic excursion in this cohort. Lack of availability of these parameters limits our ability to further phenotype the PH in the cohort, or to assess the association of co-morbidities with a potential cause of PH (pulmonary vascular resistance) or a consequence of PH (RV dysfunction). 5) Spirometry is known to over diagnose restriction [46] and in the absence of total lung capacity measurement the prevalence of restrictive lung disease could be an overestimation. 6) Lastly, the prevalence of sickle cell anemia, a condition that is associated with increased TR velocity and PH [47], was unknown, as was the prevalence of liver disease; hence, we are unable to assess the prevalence ratios related to these conditions in our cohort. 

In conclusion, PH detected by echocardiography is prevalent in AAs, more in women than in men. Besides female gender, restrictive spirometry, and obesity, other previously known risk factors for PH for the general population, such as age, pulse pressure, diabetes, obstructive lung disease, and severe left-sided valvular heart disease, were also significant determinants of PH in this AA cohort. The presence of PH portends worse outcomes in cardiopulmonary disease states that are common in the AA population. Therefore, the cardiopulmonary risk factors related to an increased prevalence of PH identified in this study may assist in the early diagnosis and management of these at-risk subjects.

## Supporting Information

Table S1 **Characteristics of Participants with or without Tricuspid Regurgitant Velocity Measurement.**
(DOCX)
