# Supplementary material for: Prevalence and Clinical Characteristics Associated with Pulmonary Hypertension in African-Americans
Source: PLoS One. 2013 Dec 16;8(12):e84264. doi: 10.1371/journal.pone.0084264 (PMC3865255; doi:10.1371/journal.pone.0084264)
Supplement: Table S1 — Characteristics of Participants with or without Tricuspid Regurgitant Velocity Measurement. (DOCX) [file pone.0084264.s001.docx]

**Table S1: Characteristics of Participants with or without Tricuspid Regurgitant Velocity Measurement***

|  | **Whole Cohort** | **Participants without TR velocity** | **Participants with TR velocity** | **p value** |
| --- | --- | --- | --- | --- |
|  | (n=5076) | (n=1794) | (n=3282) |  |
|  | Mean ± SD or % | Mean ± SD or % | Mean ± SD or % |  |
| **Age** | 54.95 ± 12.78 | 52.95 ± 12.82 | 56.05 ± 12.63 | <0.0001 |
| **Male** | 37% | 44% | 32% | <0.0001 |
| **BMI Category** | | | | |
| Normal | 14.4% | 12.9% | 15.2% | <0.001 |
| Overweight | 32.2% | 29.3% | 33.7% |  |
| Obese | 53.5% | 57.8% | 51.1% |  |
| **Smoking history** | | | | |
| Never Smoker | 68.0% | 67.8% | 68.1% | 0.036 |
| Former Smoker | 19.1% | 17.9% | 19.8% |  |
| Current Smoker | 12.9% | 14.3% | 12.1% |  |
| **Systolic BP** (mmHg) | 126.91 ± 18.24 | 127.7 ± 18.25 | 126.48 ± 18.22 | 0.023 |
| **Diastolic BP** (mmHg) | 78.73 ± 10.54 | 79.66 ± 10.66 | 78.21 ± 10.45 | <0.0001 |
| **Hypertension** | 61.6% | 62.7% | 61.0% | 0.231 |
| **Diabetes** | 26.4% | 29.7% | 24.6% | <0.0001 |
| **Coronary heart disease** | 7.9% | 7.3% | 8.3% | 0.177 |
| **Chronic lung disease** | 7.1% | 7.4% | 7.0% | 0.62 |
| **Cardiac function** | | | | |
| EF<50% | 3.2% | 3.9% | 2.8% | 0.043 |
| Mean EF (%) | 61.8 ± 7.65 | 60.95 ± 7.63 | 62.26 ± 7.63 | <0.0001 |
| LA diameter index (mm/m^2^) | 17.71 ± 2.46 | 17.23 ± 2.33 | 17.97 ± 2.49 | <0.0001 |
| LA dilation (≥4cm) | 11.97% | 10.75% | 12.63% | 0.051 |
| Severe valvular disease | 0.2% | 0.2% | 0.2% | 0.723 |
| **Pulmonary function (spirometry)** | | | | |
| Normal | 68.8% | 67.4% | 69.6% | 0.021 |
| Obstruction | 9.1% | 8.4% | 9.5% |  |
| Restriction | 22.1% | 24.2% | 21.0% |  |

*Comparison of Participants with or without measurable TR velocity. BMI: Body Mass Index; BP: blood pressure; EF: left ventricular Ejection Fraction; LA: Left Atrium; TR: Tricuspid Regurgitation
